# An *In Vitro* Microfluidic Alveolus Model to Study Lung Biomechanics

**DOI:** 10.3389/fbioe.2022.848699

**Published:** 2022-02-18

**Authors:** Vardhman Kumar, Sajeesh Kumar Madhurakkat Perikamana, Aleksandra Tata, Jiaul Hoque, Anna Gilpin, Purushothama Rao Tata, Shyni Varghese

**Affiliations:** ^1^ Department of Biomedical Engineering, Duke University, Durham, NC, United States; ^2^ Department of Orthopaedic Surgery, Duke University School of Medicine, Durham, NC, United States; ^3^ Department of Cell Biology, Duke University School of Medicine, Durham, NC, United States; ^4^ Regeneration Next, Duke University, Durham, NC, United States; ^5^ Department of Mechanical Engineering and Material Science, Duke University, Durham, NC, United States

**Keywords:** microfluidics, organ-on-a-chip, lung, microphysiological system, *in vitro* system

## Abstract

The gas exchange units of the lung, the alveoli, are mechanically active and undergo cyclic deformation during breathing. The epithelial cells that line the alveoli contribute to lung function by reducing surface tension *via* surfactant secretion, which is highly influenced by the breathing-associated mechanical cues. These spatially heterogeneous mechanical cues have been linked to several physiological and pathophysiological states. Here, we describe the development of a microfluidically assisted lung cell culture model that incorporates heterogeneous cyclic stretching to mimic alveolar respiratory motions. Employing this device, we have examined the effects of respiratory biomechanics (associated with breathing-like movements) and strain heterogeneity on alveolar epithelial cell functions. Furthermore, we have assessed the potential application of this platform to model altered matrix compliance associated with lung pathogenesis and ventilator-induced lung injury. Lung microphysiological platforms incorporating human cells and dynamic biomechanics could serve as an important tool to delineate the role of alveolar micromechanics in physiological and pathological outcomes in the lung.

## Introduction

The lung is a key organ that ensures blood oxygenation by means of respiration. Mechanical cues arising from cyclic expansion and contraction of alveoli during breathing have an important role in maintaining tissue homeostasis (Ingenito et al., 2005; Faffe and Zin, 2009; Perlman et al., 2011; Chen et al., 2014; Hsia et al., 2016; Knudsen and Ochs, 2018; Plantier et al., 2018). Conventional *in vitro* models of lung tissues frequently neglect the dynamic tissue biomechanics associated with respiration. Development of microfluidic organ-on-a-chip technology enables recapitulation of mechanical and structural aspects of the organ microenvironment, thus potentially narrowing the gap between *in vitro* and *in vivo* models (Bhatia and Ingber, 2014; Aung et al., 2016, 2020; Agrawal et al., 2017; Kumar and Varghese, 2019; Low et al., 2021). Toward this, various microphysiological platforms such as lung-on-a-chip, airway-on-a-chip, and alveolus-on-chip have been developed to model lung functions such as barrier properties, immune responses to infections, and lung pathologies such as asthma, edema, thrombosis, and lung cancer progression (Huh et al., 2010, 2012; Stucki et al., 2015, 2018; Benam et al., 2016a, 2016b, 2017; Jain et al., 2018; Park et al., 2018; Felder et al., 2019; Elias-Kirma et al., 2020; Ishahak et al., 2020; Jimenez-Valdes et al., 2020; Khalid et al., 2020; Mejías et al., 2020; Nawroth et al., 2020; Shrestha et al., 2020, 2021; Zamprogno et al., 2021).

Lung distention occurs in three dimensions (3D) as a result of not only the alveolar air pressure but also the difference between the alveolar air pressure and the pleural fluid pressure, the transpulmonary pressure. Model systems incorporate such dynamic mechanical movements associated with breathing using deformable membranes subjected to dynamic pressure (Guenat and Berthiaume, 2018). While most *in vitro* studies and microphysiological systems incorporating dynamic stretch have been limited to generating in-plane strain or uniaxial strain in the 5–12% range reported *in vivo* (Birukov et al., 2003; Guenat and Berthiaume, 2018; Nossa et al., 2021; Sznitman, 2021), recent studies have delved into incorporating out-of-plane stretching to mimic the 3D movements of the alveoli during respiration (Stucki et al., 2018; Doryab et al., 2021a; Huang et al., 2021; Zamprogno et al., 2021). These advances offer unique *in vitro* tools to study the micromechanical changes such as the strain heterogeneity that develops as a result of the out-of-plane stretch and its subsequent role in lung physiology and pathology. Although the precise strain profile that develops in the lung during respiration is yet to be accurately mapped, it is well established that not just different regions of the lung but even a single alveolus experiences varying strains during respiration (Roan and Waters, 2011). These spatially varying mechanical cues have been shown to be associated with several lung conditions and vulnerabilities. For example, regions with intrinsically high strains—such as in aerated alveoli next to fluid-filled alveoli—are prone to experiencing increased strains during mechanical ventilation, thus predisposing them to ventilator-induced lung injury (VILI) (Perlman et al., 2011; Smith, 2016). Apart from such spatial variations in strain during breathing, progressive changes in local tissue mechanics (e.g., changes in compliance) are key characteristics of various respiratory disorders such as pulmonary fibrosis and emphysema (Ingenito et al., 2005; Faffe and Zin, 2009; Plantier et al., 2018; Hurtado et al., 2020).

In this study, we report a microfluidic alveolar tissue model that mimics dynamic out-of-plane stretching akin to alveolar inflation and deflation associated with breathing. The device consists of a fluidic and pneumatic layer separated by a thin polydimethylsiloxane (PDMS) membrane, which was functionalized by covalent conjugation of collagen-I to enable cell culture. We characterized the heterogeneous strain experienced by the membrane as a result of breathing-like movements by using computational analyses and examined the effect of spatial heterogeneity on cell alignment. Employing this platform, we also examined the effect of biomechanics on the function of alveolar epithelial cells. Specifically, we studied the effect of breathing-like movements on surfactant production by alveolar epithelial cells and how it is affected by heterogeneous strain and altered matrix compliance. Furthermore, we incorporated transpulmonary pressure into the device to have the membrane deform in response to a combination of pneumatic and hydrostatic pressure. Using this approach, we determined the applicability of this platform to model ventilator-induced lung injury (VILI). Finally, we also examined the potential of the platform to support the culture of human primary alveolar epithelial type 2 (AT2) cells and AT2 cells derived from human induced pluripotent stem cells (hiPSC).

## Methods

### Photolithography of the Master Mold

Silicon wafers (UniversityWafers) were used for patterning arrays of 200-μm-wide and 100-μm-high channels by using SU-8 photolithography. Briefly, 4 ml of SU-8 100 photoresist was spin-coated on a cleaned wafer at 3,000 rpm/s for 30 s. The wafer was baked at 65°C for 10 min, followed by 95°C for 30 min. The wafer was then exposed to 365 nm wavelength light through a custom photomask designed in AutoCAD and baked at 65°C for 1 min and 95°C for 10 min. The wafer was then rinsed with SU-8 developer to remove the undeveloped photoresist. The resulting master mold was cleaned with isopropanol followed by water and stored until use.

### Device Fabrication, Assembly, and Operation

**FIGURE 1:**
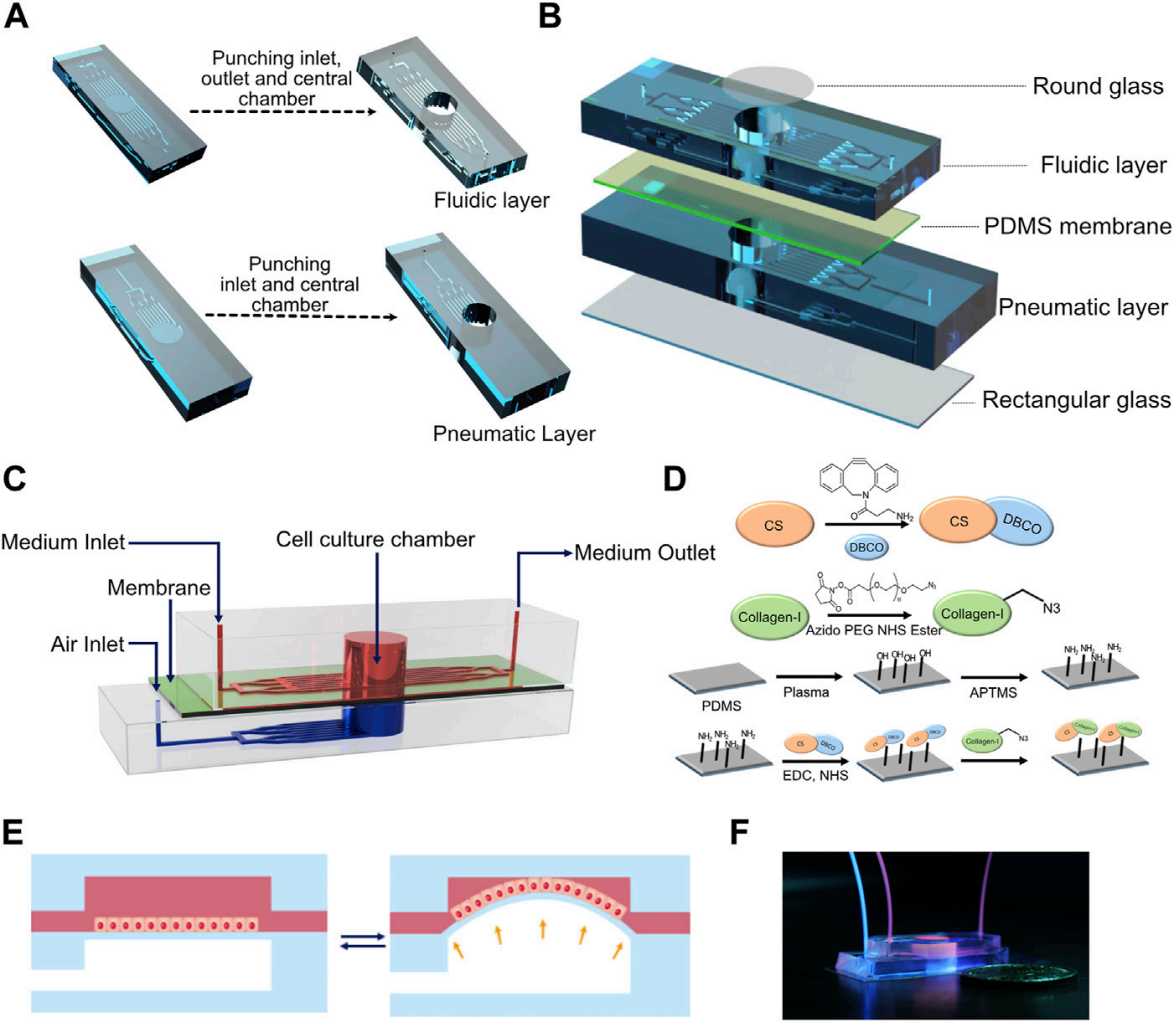
**(A)** Computer-aided design (CAD) of the fabrication process of fluidic and pneumatic layers with microchannels. **(B)** Schematic of device assembly showing the different layers of the device in an exploded view. **(C)** Schematic depicting the assembled device. **(D)** Reaction scheme used for collagen functionalization of the PDMS membrane. **(E)** Schematic of breathing-like movements within the device. **(F)** Digital photograph of the device showing the media/cell chamber (red) and air chamber (blue).

To fabricate the device, a 10:1 (base:crosslinker) polydimethylsiloxane (PDMS) (Sylgard 184, Dow Inc.) precursor solution containing the crosslinker was poured onto the master mold and allowed to cure for 2 h at 60°C. Post curing, PDMS was cut around the channels and 8 mm diameter holes were punched in the top fluidic layer and bottom pneumatic layer to create cell culture chambers and pneumatic chambers, respectively (Figure 1A). The same channel dimensions were used for both fluidic and pneumatic channels. One-mm-diameter inlet and outlet were punched in the fluidic layer, and a 1-mm-diameter inlet was punched in the pneumatic layer of the device (Figure 1A). PDMS membranes were prepared by curing the precursor solution between two glass slides separated using stainless steel spacers with a thickness of 250 μm. The cell culture chamber was capped on the top by a glass coverslip using either double-sided tape (for reversible adhesion) or plasma bonding (for irreversible adhesion). The fluidic microchannels were capped at the base by plasma bonding the PDMS membrane to the fluidic layer. Separately, the pneumatic chamber and the pneumatic channels were capped at the bottom by plasma bonding a rectangular coverslip to the pneumatic layer (Figure 1B). The fluidic and pneumatic layers were assembled using double-sided tape to get a reversible adhesion between the two layers. This enabled easy disassembly of the device for imaging. The PTFE tubing from the programmable air pump (Elveflow) was connected to the inlet of the pneumatic layer, while the inlets of the fluidic layer were fed through syringes loaded onto a syringe pump (Harvard Apparatus) at a rate of 50 μl/h unless stated otherwise. For all experiments involving breathing-like motions, sinusoidal pressure waveforms with a minimum and maximum at 0 and 50 mbar, respectively, at a frequency of 0.5 Hz were used unless stated otherwise.

### Computational Modeling to Determine Strain Fields

The breathing movements of the membrane were modeled in COMSOL. The membrane was modeled as a cylindrical disc made from PDMS. A Mooney–Rivlin hyperelastic model (Rivlin, 1948) was used to describe the membrane:

$$W_{s}=\overset{n}{\underset{i,j=0}{\sum }}C_{i,j}{\left(I_{1}-3\right)}^{i}{\left(I_{2}-3\right)}^{j}+\frac{1}{2}K{\left(J_{el}-1\right)}^{2},$$ (1)

where $I_{1}$ and $I_{2}$ are the first and second invariant of the left isochoric Cauchy-Green deformation tensor, K is the bulk modulus, $J_{el}$ is the elastic Jacobian, and $C_{i,j}$ are material parameters.

Two-parameter Mooney–Rivlin material parameters for the PDMS membrane of 10:1 base to crosslinker were approximated from Yoon et al. (2010). The values used are listed in Supplementary Table S1. Zero displacement boundary condition was applied at the curved surface of the cylindrical membrane, while load (resulting from air pressure) was applied at the base of the membrane. Displacement field was used to calculate the resulting radial and circumferential strain as a function of radial distance from the center of the membrane (Winkler et al., 2014).

$$Radial\;Strain=\;\frac{\left|\left(x_{2}^{s}-x_{1}^{s}\right)+\left(y_{2}^{s}-y_{1}^{s}\right)+\left(z_{2}^{s}-z_{1}^{s}\right)\right|\;-|\left(x_{2}-x_{1}\right)+\left(y_{2}-y_{1}\right)+\left(z_{2}-z_{1}\right)|}{|\left(x_{2}-x_{1}\right)+\left(y_{2}-y_{1}\right)+\left(z_{2}-z_{1}\right)|}\;$$ (2)

$$Circumferential\;Strain=\frac{2\pi r^{s}-2\pi r}{2\pi r},$$ (3)

where $\left(x_{1},y_{1},z_{1}\right)$ and $\left(x_{2},y_{2},z_{2}\right)$ are coordinates of two arbitrary points on the membrane before stretching and $\left(x_{1}^{s},y_{1}^{s},z_{1}^{s}\right)$ and $\left(x_{2}^{s},y_{2}^{s},z_{2}^{s}\right)$ are the coordinates of the points after stretching. Similarly, r and $r^{s}$ are the radial distance of a point from the center of the membrane before and after stretching, respectively.

### Synthesis of Chondroitin Sulfate–Dibenzocyclooctyne and Collagen Azide

Five hundred milligrams of chondroitin sulfate (CS) (Alfa Aesar, J60341) was dissolved into 60 ml DI water. To this, 35 ml of dimethyl sulfoxide (DMSO) was added, followed by 287.5 mg of 1-ethyl-3-(3-dimethylaminopropyl) carbodiimide hydrochloride (EDC.HCl) (TCI Chemical, D1601). Then 172.6 mg of *N*-hydroxy succinimide (NHS) (Sigma, 130672) and 138.15 mg of dibenzocyclooctyne–amine (DBCO-amine) (Click Chemistry Tools, A103) were dissolved in 5 ml of DMSO and were added to the reaction mixture at 15-min intervals. The reaction was continued for 24 h under constant stirring. The reaction mixture was then dialyzed against water for 4 days and freeze-dried to obtain DBCO-conjugated chondroitin sulfate (CS-DBCO). The product was stored at −20°C until use. The product was characterized by a combination of Fourier transform infrared spectroscopy (FTIR) and proton nuclear magnetic resonance (^1^HNMR) spectroscopy. The peaks in attenuated total reflection (ATR) FTIR spectra at 1,612 cm^−1^ due to C=O stretching and 1,559 cm^−1^ due to N–H deformation in the CS spectrum were shown to be shifted to 1,648 and 1,569 cm^−1^ in the CS-DBCO spectrum, respectively, indicating the presence of new amide bonds formed via the reaction between the carboxylic acid group of CS and the amine group of DBCO. In addition, peaks were observed at 1,480 and 1,441 cm^−1^, which represented the aromatic C=C stretching in the rings of DBCO (Supplementary Figure S1). The successful conjugation of DBCO to CS was further confirmed by ^1^HNMR where the spectrum showed the appearance of aromatic protons from DBCO at 7.34–7.44 ppm. The degree of DBCO conjugation was calculated by taking the ratio of area under the curve for the aromatic protons of DBCO at 7.34–7.44 ppm to the –CH_3_ protons of the –NHCOCH_3_ groups of CS at 1.9 ppm and found to be 39 ± 2% with respect to the dimeric sugar unit of CS (Supplementary Figure S2).

For collagen azide, 1 mg of collagen type I (Corning, 354236) and 500 μl of azide-polyethylene glycol-NHS) (Click Chemistry Tools, AZ103) were dissolved in 10 ml PBS each. The reaction was performed by mixing the solutions under constant stirring for 3 h at 0–4°C. The reaction mixture was then dialyzed against water at 4°C for 3 days. The product was freeze-dried and stored at -20°C until use. The product was characterized via FTIR spectroscopy where the spectra revealed a sharp peak at 2,141 cm^−1^ characteristic to the N≡N stretching frequency of the azide functional group (Supplementary Figure S3).

### Collagen Functionalization of the Membrane

The PDMS membrane in the bonded devices was treated with corona treater (ETP, BD-20AC) for 1 min to plasma-activate the surface. Approximately 100 μl of 2% (v/v) (3-aminopropyl)-trimethoxysilane (APTMS) (Sigma, 281778) in 100% ethanol was introduced into the cell culture chamber, and the devices were incubated for 60 min at room temp and washed with distilled water. Meanwhile, 5 mg of CS-DBCO was dissolved in 1 ml of PBS and reacted with 5 mg of EDC for 15 min, followed by 2.1 mg of NHS to convert CS-DBCO into CS-DBCO-NHS ester. The devices were incubated with this activated CS-DBCO-NHS ester at 37°C for overnight to covalently immobilize CS-DBCO on the PDMS surface. The next day, the devices were washed 2–3 times with distilled water, and 10 μg/ml of collagen-azide solution in PBS was introduced into the devices. The devices were incubated overnight to immobilize collagen onto the PDMS surface via a strain-promoted azide–alkyne cycloaddition (SPAAC) reaction between the azide group of collagen zide and the DBCO group of surface-bound CS-DBCO. The devices were washed with PBS before culturing cells.

### Isolation and Culture of Human Primary AT2 Cells

Healthy human lungs were procured through the BioRepository and Precision Pathology Center at Duke University in accordance with institutional procedures (Duke University Pro00082379—“Human Lung Stem Cells”; exempt research as described in 45 CFR 46.102(f), 21 CFR 56.102(e) and 21 CFR 812.3(p) which satisfies the Privacy Rule as described in 45CFR164.514). Human lung dissociation was performed as described previously (Zacharias et al., 2018). Briefly, approximately 2 g human lung tissue was cut into small pieces and incubated with 30 ml of enzyme mixture (collagenase type I (Gibco, 17100–017): 1.68 mg/ml, dispase (Corning, 354235): 5 U/ml, DNase (Thermo Fisher Scientific, 10104159001): 10 U/ml) at 37°C for 1 h with continuous rotation. The cells were filtered through a 100-μm cell strainer and rinsed with DMEM/F12 containing 10% FBS and anti-anti through the strainer. The sample was centrifuged at 450 g for 10 min, and the cell pellet was resuspended in red blood cell lysis buffer for 10 min, washed with DMEM/F12 containing 10% FBS, and filtered through a 40-µm strainer. Total cells were centrifuged at 450 g for 5 min at 4°C and the cell pellet was processed for alveolar type 2 cell (AT2s) purification. AT2s were isolated by magnetic-activated cell sorting (MACS) or fluorescence-activated cell sorting (FACS)-based protocols as described previously (Katsura et al., 2020). Approximately 2–10 million total human lung cells were resuspended in MACS buffer and incubated with Human TruStain FcX (Biolegend, 422032) for 15 min at 4°C, followed by incubation with HTII-280 (Terrace Biotech, TB-27AHT2-280) (1:60 dilution) antibody for 1 h at 4°C. The cells were washed twice with MACS buffer and incubated with anti-mouse IgM microbeads for 15 min at 4°C. The sample was loaded into the LS column (Miltenyi Biotec), and cells were collected magnetically. For FACS based purification of human AT2s, the total lung cell pellets were resuspended in MACS buffer and the EpCAM-positive population was purified using microbeads according to the manufacturer’s instructions (Miltenyi Biotec, 130-061-101). CD326-positive cells were stained with HTII-280 and LysoTracker (Thermo Fisher Scientific, L7526) at 37°C for 25 min, followed by incubation with secondary antibody Alexa anti-mouse IgM-488 (Thermo Fisher Scientific, 10680) for 10 min at 37°C, washed twice and sorted using a FACS Vantage SE and SONY SH800 S. The cells were expanded as alveolospheres in growth factor reduced Matrigel (Corning, 354230) as described previously (Katsura et al., 2020), using Advanced DMEM/F12 medium containing 10 μM SB431542 (Abcam, 120163), 3 μM CHIR99021 (Tocris, 4423), 1 μM BIRB796 (Tocris, 5989), 10 μM Y27632 (Selleckchem, S1049) (for first 4 days of culture), 50 ng/ml Human EGF (Gibco, PHG0313), 10 ng/ml Human FGF10 (Biolegend, 559304), 5 μg/ml Heparin (Sigma, H3149), 1X B27 supplement (Thermo Fisher Scientific, 17504044), 1X Antibiotic-Antimycotic (Thermo Fisher Scientific, A5955), 15 mM HEPES, 1X Glutamax (Thermo Fisher Scientific, 35050061), and 1.25 mM N-Acetyl-l-Cysteine (Sigma, A9165).

### Culture of Human Induced Pluripotent Stem Cell (hiPSC)-Derived AT2 Cells (iAT2 Cells)

IPSCs-derived human alveolar type 2 cells (iAT2s) (differentiated from the SPC2-ST-B2 iPSC line) were expanded as alveolospheres in growth factor reduced Matrigel (Corning, 354230) as described elsewhere (Jacob et al., 2019). The iAT2s were maintained in IMDM medium containing 25% Ham’s F12 (Cellgro, 10–080-CV), 1% B27 supplement, 0.5% N2 supplement, 0.05% BSA (Invitrogen, 15260037), 200 ng/ml Primocin (Invivogen, NC9141851), 1X GlutaMAX, 50 μg/ml ascorbic acid (Sigma, A4544), 0.45 mM monothioglycerol (Sigma, M6145), 3 μM CHIR99021, 10 ng/ml rhKGF (R&D, 251-KG-010), 50 nM Dexamethasone (Sigma, D4902), 10 μM Y27632 (for first 3 days of culture), 0.1 mM 8-bromoadenosine 3′,5′-cyclic monophosphate sodium salt (Sigma, B7880), and 0.1 mM 3-isobutyl-1-methylxanthine (Sigma, I5879).

### Culture of MLE-12 and H441 Cell Lines

MLE-12 cells were maintained in HITES medium consisting of DMEM/F12 (ATCC, 30-2006), 2% fetal bovine serum (FBS), 0.005 mg/ml insulin, 0.01 mg/ml transferrin (Fitzgerald, 31C-CH1026), 30 nM sodium selenite (Santa Cruz, 253595), 10 nM hydrocortisone (Sigma, H0888), 10 nM β-estradiol, 10 mM HEPES (Gibco, 15630106), 2 mM l-glutamine (Gibco, 25030081), and 1% Pen-Step (Gibco, 15140122). H441 cells were maintained in RPMI-1640 (ATCC, 30-2001) medium supplemented with 10% FBS and 1% Pen-Strep.

### Cell Seeding Within the Device

For seeding hAT2s and iAT2s into the devices, alveolosphere dissociation was carried out by incubating the cultures in 2 mg/ml dispase for 30 min to release the alveolospheres from the Matrigel matrix. This was followed by centrifugation at 200 g for 4 min and resuspension of the alveolospheres in 0.05% Trypsin-EDTA for 5 min. The cell suspension was centrifuged at 300 g for 5 min and the cells were resuspended in their medium before counting. For cell lines, the cells were detached from the culture plate using 0.25% trypsin–EDTA, centrifuged, and resuspended in medium before counting.

Single cells were suspended in their corresponding medium at a concentration of 2 × 10^5^ cells/ml; 100 μl of the cell suspension was perfused into the fluidic chamber of the device. The cell-loaded devices were left undisturbed (2 h for cell lines and 6 h for hAT2s and iAT2 cells) to allow the cells to adhere to the membrane before they were connected to the syringe pump to start the perfusion. The cells were cultured in a submerged condition within the device. A tubing was connected from the fluidic outlet into an Eppendorf tube to collect the perfusate. Twenty-four hours after cell seeding, the air pump was connected to the pneumatic inlet of the devices to induce breathing-like motion.

### Immunofluorescence Staining and Quantification

The fluidic layer was separated from the pneumatic layer, and the cover slip was removed from the top. The cells were washed three times with PBS and fixed with 4% PFA for 15 min. The cells were washed again with PBS and treated with a permeabilization solution for 15–20 min (PBS/0.1% Triton-X). After permeabilization, the cells were blocked using 5% normal donkey serum in 1% BSA. Cells were then treated with anti-prosurfactant protein C primary antibody (Sigma, AB3786) for overnight at 4°C (1:150). The next day, the cells were washed 3 times with PBS and incubated with the secondary antibody (anti-rabbit Alexa Flour 647, 1:200) for 1 h. The staining solution was then removed, Hochest (1:1,000) was added, and the mixture was incubated for 4 min. Finally, cells were washed and imaged using a Keyence BZ microscope. Mean fluorescence intensity was quantified using ImageJ. Another set of cultures were stained with phalloidin (1:200) for 1 h and with Hochest (1:1,000) for 4 min. The cells were washed, and images of various locations within the membrane were acquired. OrientationJ plugin (Püspöki et al., 2016) within ImageJ was used to analyze the images.

### Surfactant Protein-A ELISA

For Surfactant Protein-A (SP-A) ELISA experiments, devices were seeded with H441 cells, which were allowed to grow to confluence for 3 days before exposing them to breathing-like motions. Just before the start of the experiments, the outlet reservoir was emptied, and the flow rate was reduced to 5 μl/h to concentrate the secreted surfactant. After 24 h of breathing, it was visually confirmed under a microscope that the cell layer had not detached during the experiment. Perfusate collected in the outlet reservoir was used for surfactant protein-A ELISA using the SP-A kit (Biovender, RD191139200R) according to the manufacturer’s instructions.

### Determining Concave Volume and Compliance Curves

The concave volume of the membrane in response to applied air pressure (0–50 mbar) was calculated using COMSOL. The side view of the computational model of the membrane in stretched position was projected onto a 2D plane. The boundary of the membrane was traced to obtain the spatial points. A 6-order polynomial curve was fitted onto the points to obtain the equation for the curve. The volume encompassed was calculated by integrating the equation in three dimensions using Wolfram Alpha. This was done for membranes of different thicknesses 250 μm and 1,000 μm subjected to different pressures. The obtained volumes were plotted against the corresponding applied pressure to obtain the compliance plot.

### Transpulmonary Pressure Setup

To introduce hydrostatic pressure, the outlet of the fluidic microchannels was blocked using knotted PTFE tubing. A 10-ml syringe was filled with cell culture medium and its plunger was removed to expose the media to the atmosphere. A lid was placed at the end of the syringe to reduce the chances of contamination. Tubing was connected from the syringe to the inlet of the fluid microchannels of the device. To generate positive hydrostatic pressure of 50 mbar, the syringe was placed 50 cm above the height of the device throughout the experiment.

### Live-Dead Analysis

A viability/cytotoxicity kit (Thermo Fisher Scientific, L3224) was used to determine cell viability. Briefly, media was aspirated from the fluidic layer of the device, and the cells were washed with PBS. Then 100 μl of solution containing 0.05% calcein AM and 0.2% ethidium homodimer-1 was introduced into the device. The cells were incubated in the solution for 30 min before washing them with PBS and imaging them.

### Statistics

**FIGURE 2:**
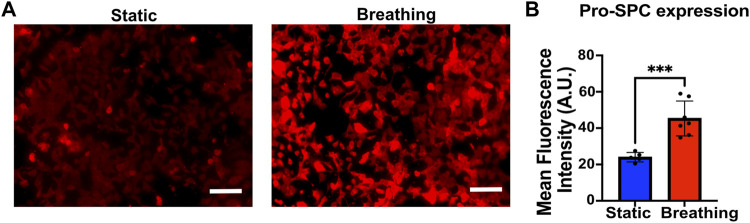
**(A)** Immunofluorescent staining of Pro-SPC (red) of MLE-12 cells cultured under static and breathing conditions. **(B)** Quantification of the fluorescent intensity reveals that cells exposed to breathing-like motions express significantly higher pro-SPC. Each data point is an average of fluorescence intensity measurement from 6 to 11 images per independent device (N = 5 for static and N = 7 for breathing). Scale: 100 μm.

Statistical analysis was performed on GraphPad Prism 9. An unpaired *t*-test was used to compare the mean fluorescent intensity in Figure 2 and Figure 6; the cell shape in Figure 6 and the SP-A levels in Supplementary Figure S5. For comparison of mean fluorescence intensity of cells cultured on 250- and 1,000-μm-thick membranes across center and edge, respectively, 2-way repeated measures ANOVA followed by multiple comparisons was used, with “center” and “edge” values being the repeated measures for each device. N ≥ 3 independent devices per experimental group were used across all experiments. *p*-values < 0.05 were considered statistically significant.

## Results and Discussion

### Device Fabrication and Cell Culture

The device consists of a bottom pneumatic layer and a top fluidic layer separated by a thin PDMS membrane (Figure 1C). The top layer consists of an inlet that diverges into an array of microchannels leading to a cylindrical cell culture chamber. This chamber is lined with a thin membrane at the base. Another array of microchannels connects the cell culture chamber to the device outlet. These channels are used for cell seeding and medium perfusion. In the bottom layer, the inlet diverges into an array of microchannels that lead to a cylindrical chamber whose ceiling is formed by the thin membrane and whose base is capped by a cover glass. The bottom layer is devoid of any outlets so that the device can be subjected to a pressure waveform to induce breathing-like movements. The PDMS membrane facing the fluid side was functionalized with collagen type I to promote cell attachment (Figure 1D). When subjected to a positive pressure in the bottom layer, the membrane concaves downward, which induces stretching of the membrane (Figure 1E). Applying a pressure waveform *via* the air pump facilitates cyclic stretching of the membrane, thus mimicking the breathing movements of alveoli (Supplementary Movie S1). Digital images of the device and of the setup are shown in Figure 1F and Supplementary Figure S4, respectively. Cells were perfused into the device *via* the inlet channel and allowed to adhere before introducing continuous perfusion of medium. The cells were cultured for 24 h before subjecting them to breathing-like movements. The device supported culture of alveolar epithelial cell lines, human primary alveolar epithelial cells (hAT2s) and human induced pluripotent (hiPSC)-derived alveolar epithelial cells (iAT2s).

### Effect of Breathing-Like Motion on Surfactant Production

To study the effect of breathing-like motion-mediated active mechanical cues on alveolar cell functions, the epithelial cells within the device were subjected to cyclic stretching. The MLE-12 cells were subjected to breathing-like motions for 24 h, following which they were fixed and stained for surfactant protein-C (pro-SPC). Cells that were exposed to breathing-like motion expressed significantly higher levels of pro-SPC than cells cultured in static conditions (Figures 2A, B). Higher surfactant production in breathing cultures was further confirmed by H441 cells. As shown in Supplementary Figure S5, the cells subjected to dynamic breathing showed more secreted surfactant protein-A (SP-A). These results are in line with previous reports which showed increased surfactant production in response to cells subjected to stretching (Sanchez-Esteban et al., 1998; Torday and Rehan, 2002). The effect of mechanical cues on surfactant production has also been established in *in vivo* studies where mechanical ventilation-induced stretch has been reported to increase surfactant production in rat lungs (Martinez et al., 2004).

### Breathing-Induced Heterogeneous Strain Profile and Its Effect on Cell Shape

**FIGURE 3:**
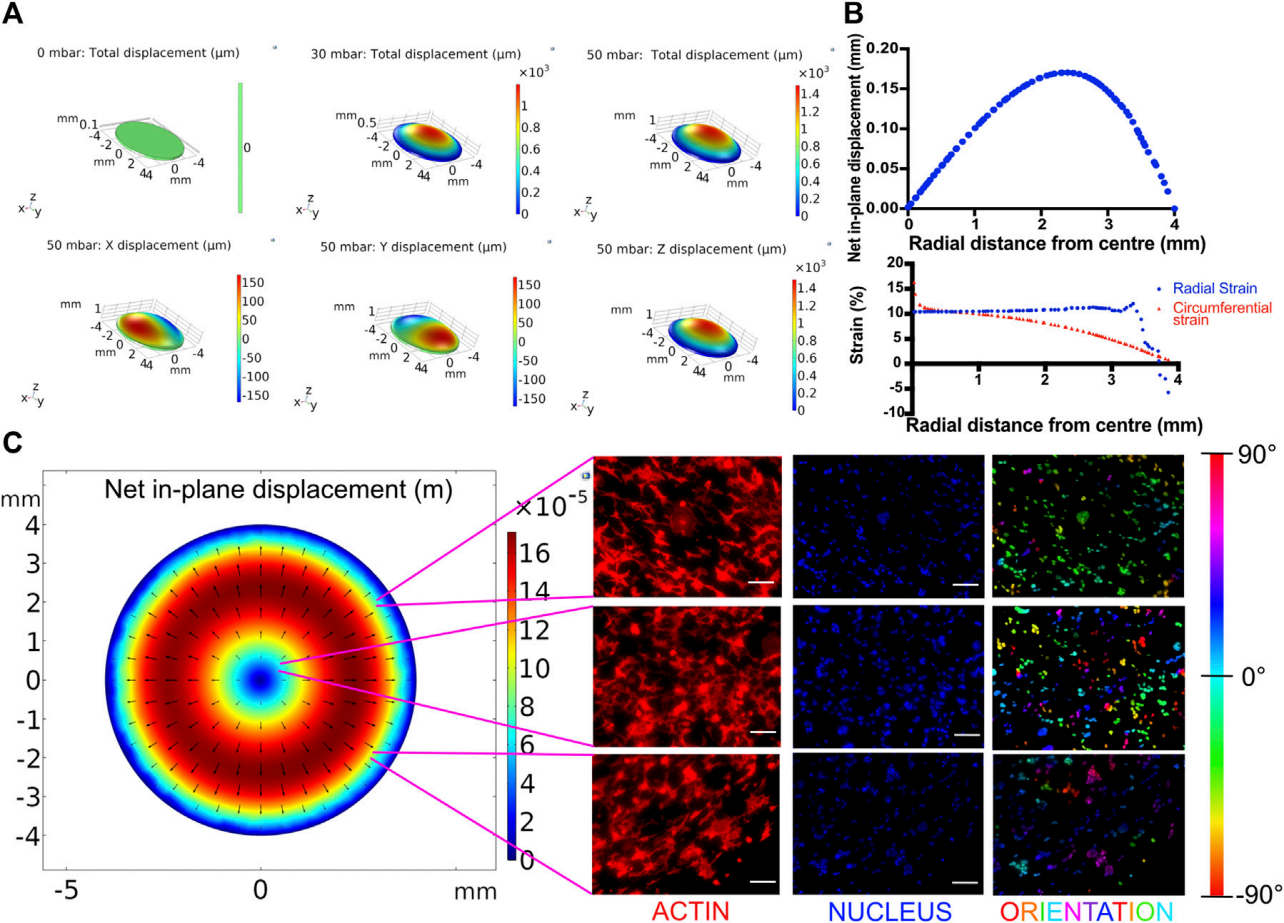
**(A)** Displacement profiles of the breathing membrane subjected to various peak pressures. **(B)** In-plane displacement, radial strain and circumferential strain profile of the cell culture membrane at 50 mbar. **(C)** Effect of spatial strain heterogeneity of the cell culture substrate on cell alignment. Left: COMSOL computed in-plane displacement of the cell culture membrane. Right: Cellular alignment of MLE-12 cells was visualized by phalloidin (first column), and nucleus (second column) staining and cellular alignment at different locations in breathing devices was quantified using nuclear orientation (third column) where color of the nuclei in the third column represents their angle of alignment. Cells at the center of the membrane align randomly while those away from center align perpendicular to the radius. Scale: 100 μm.

While dynamic stretching was found to promote surfactant production overall, we next analyzed the out-of-plane stretching-induced heterogeneous strain profile and its effect on the cells. Toward this, the membrane deformation during cyclic stretch was modeled in COMSOL as detailed in the experimental section. The effect of applied pressure on the membrane displacement field was determined by mapping the strain profile (Figure 3A). Using the displacement field, radial and circumferential strains were calculated at a peak pressure of 50 mbar. This pressure was chosen as it falls within the range of air pressure in the human lung during breathing and also results in physiological levels of strain (Roan and Waters, 2011). At this pressure, the in-plane displacement increases with increasing radial distance from the center (r) and gradually decreases, peaking at around r = 2.5 mm (Figure 3B). The circumferential strain was found to gradually decrease from 17 to 0% with increasing radial distance from the center. The radial strain largely remains constant at around 10% until a sharp decline after r > 3 mm due to the membrane being constricted at the edges (Figure 3B). MLE-12 cells on the membrane responded to the radial strain heterogeneity and exhibited a differential alignment. Specifically, at the center of the membrane, the cells were aligned randomly, while away from the center, they were aligned perpendicular to the direction of the major strain (i.e., the direction of radial displacement) (Figure 3C). In addition to epithelial cells, we have also examined the effect of strain heterogeneity on NIH 3T3 cells as fibroblasts are known to be highly sensitive to mechanical cues, including cyclic strain (Neidlinger-Wilke et al., 2002). Akin to epithelial cells, the fibroblasts displayed strain profile-dependent cell alignment, where cells away from the center aligned perpendicular to the direction of major strain, while those at the center were randomly aligned (Supplementary Figure S6). The strain-dependent alignment was more pronounced in the case of fibroblasts than in the epithelial cells, which is most likely attributed to the propensity of fibroblasts to polarize in cultures and acquire elongated morphology.

### Modeling Matrix Compliance

**FIGURE 4:**
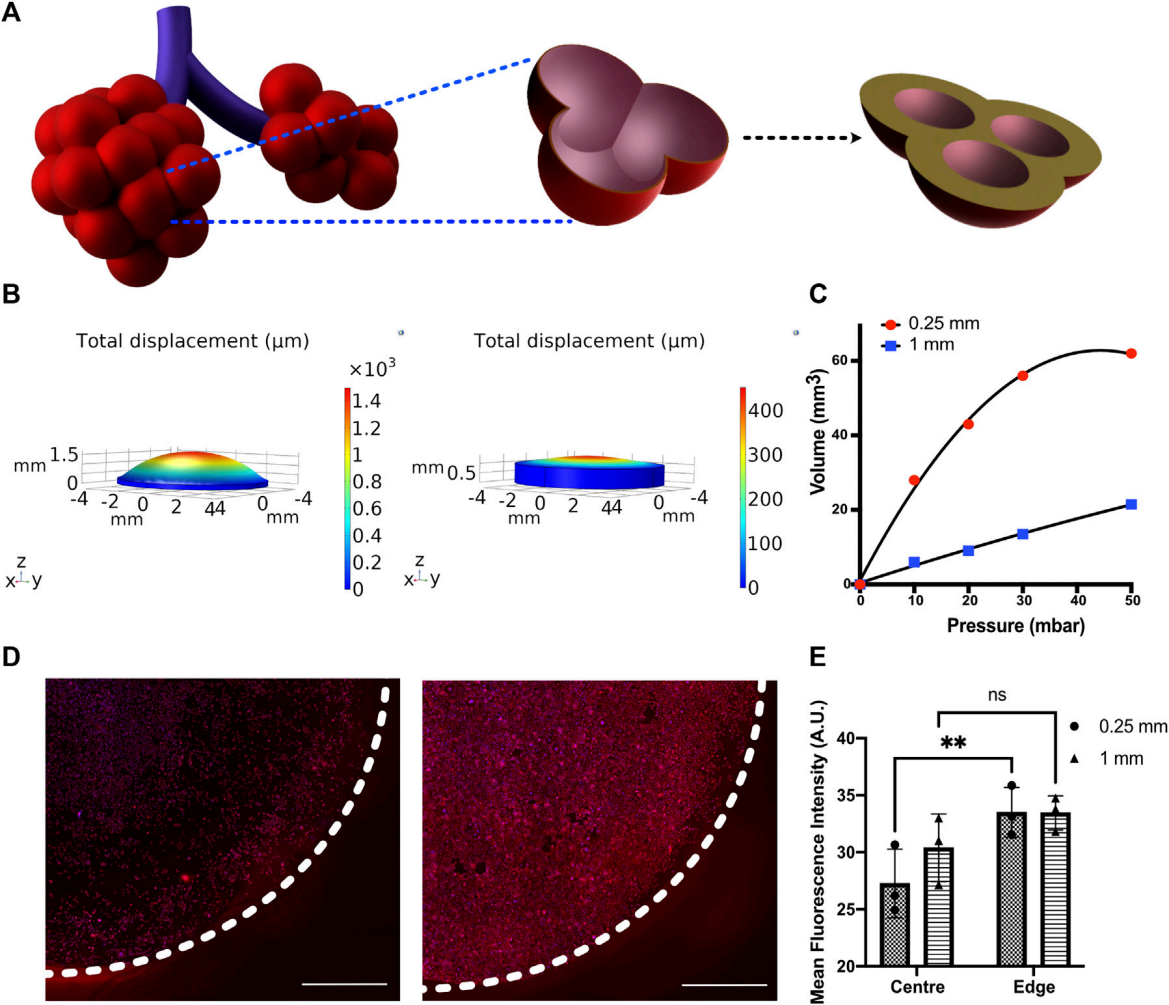
**(A)** Schematic depicting the change in alveolar wall thickness as a result of interstitial wall thickening. **(B)** Displacement profiles of membranes with 250 and 1,000 μm thickness subjected to 50 mbar pressure **(C)** Pressure–volume compliance curves for membranes with 250 and 1,000 μm thickness when subjected to a breathing-like motion **(D)** Immunofluorescent staining for Pro-SPC (red) and nucleus (blue) in MLE-12 cells cultured on 250-μm (left) and 1,000-μm (right)-thick membranes and **(E)** its quantification at center (r < 3 mm) and edge (3 mm < r < 4 mm) of the device. N = 3 where each data point is an average of fluorescence intensity measurement from 7 to 12 images per independent device. White-dotted line represents the boundary of the membrane Scale: 1 mm.

Changes in tissue compliance are a key characteristic of various lung diseases. For example, diseases like pulmonary fibrosis are characterized by alveolar wall thickening due to excessive accumulation of extracellular matrix and an increase in tissue stiffness, resulting in lowered compliance (Figure 4A). We have modeled the lowered tissue compliance by increasing the thickness of the membrane from 250 to 1,000 μm. With increasing thickness, the membrane will offer higher resistance to the same pressure profile than a 250-μm membrane, which results in less expansion/contraction and thereby a different breathing pattern (Supplementary Movie S2). The pulmonary compliance, which is expressed as the change in lung volume in response to a change in pressure ( $\frac{\text{Δ}V}{\text{Δ}P}$ ), was calculated by computationally measuring the changes in the concave volume of the membrane as a function of pressure during breathing-like movements (Figures 4B,C). We chose a membrane thickness of 250 and 1,000 μm as they offer ∼2.8 fold difference in volume at peak pressure, which is in the range of volume differences observed between healthy and fibrotic human lungs (Harris, 2005).

We examined the membrane compliance-mediated strain profile for an applied peak pressure of 50 mbar. As expected, the high compliance membrane (i.e., 250-μm membrane) encountered a significantly higher strain heterogeneity than the low compliance membrane (1,000 μm membrane). This is attributed to the lower resistance imposed by the 250 μm membrane, permitting higher deformation (Figure 4B). To examine the effect of cell response to the altered matrix compliance, MLE-12 cells were cultured in the device with membranes of thickness 250 μm and 1,000 μm and analyzed after subjecting them to 24 h of breathing-like motion. A significant difference in cell number was observed between the two, with the 1,000-μm membrane that experiences lower strains showing the presence of more cells (Figure 4D). Furthermore, the strain-dependent spatial variation in cell number was prominently observed on the 250-μm membrane, where the strain varies spatially over a wider range. Specifically, areas with high displacement exhibited lower cell numbers. On the contrary, such regional differences in cell number were not observed in the 1,000-μm membrane. Concomitant with these findings, spatial differences in surfactant production were also observed between the two conditions, as evident from the quantification of pro-SPC immunofluorescence intensity (Figure 4D).

### Modeling Transpulmonary Pressure and Ventilator-Induced Lung Injury

**FIGURE 5:**
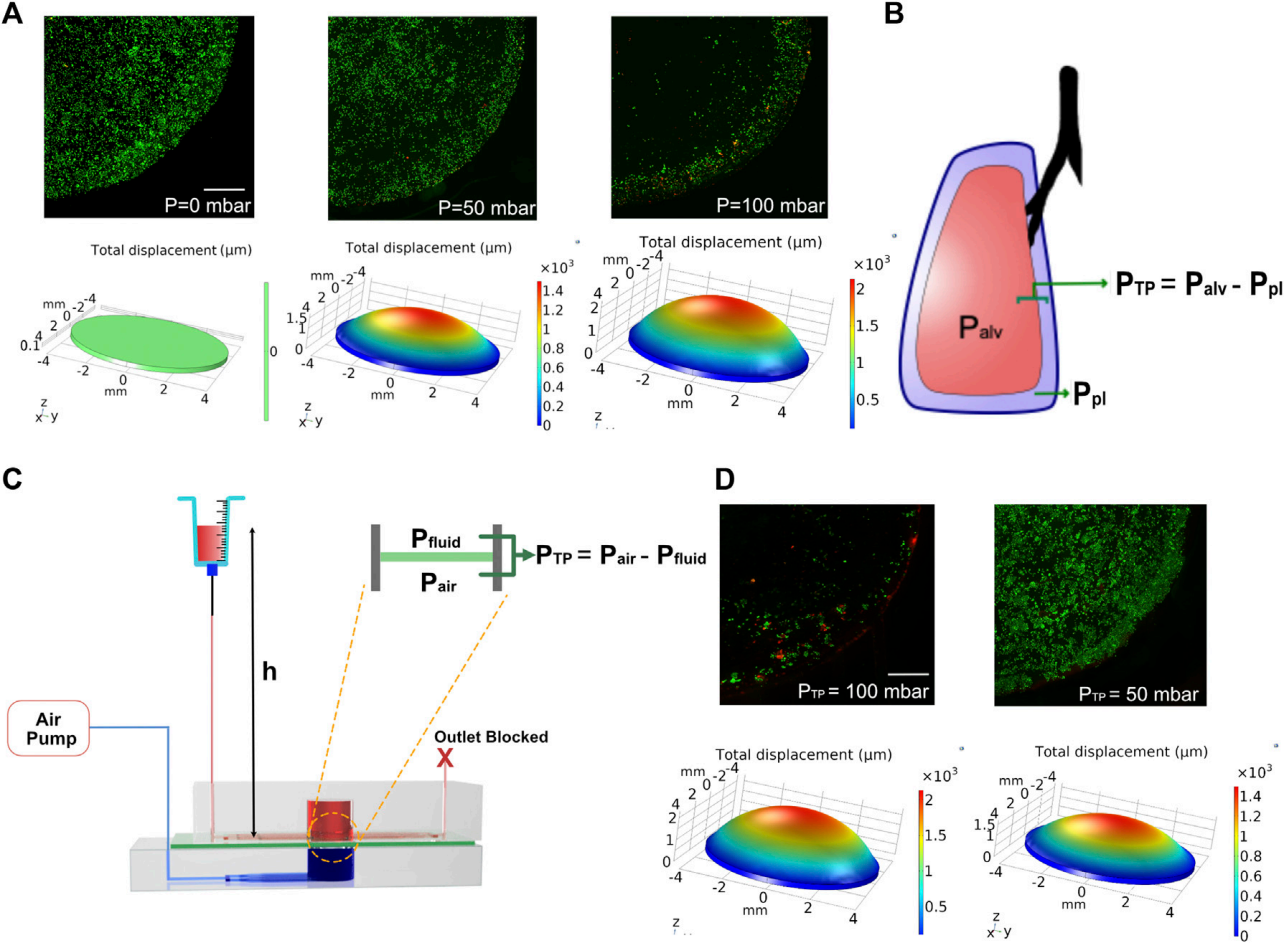
**(A)** COMSOL modeling and corresponding cell experiments for studying the effect of volutrauma as a result of applied air pressure of 0 mbar, 50, and 100 mbar. Higher air pressure leads to higher concave volume. Viability of MLE-12 cells was determined using live-dead assay. Live cells are stained green while the dead cells are stained red. **(B)** Schematic depicting transpulmonary pressure in a lung. P_TP_, P_alv_, and P_pl_ are transpulmonary pressure, air pressure in the alveoli and pleural pressure, respectively. **(C)** Schematic showing the modeling of transpulmonary pressure in the device. P_air_ and P_fluid_ represent the air pressure and hydrostatic pressure in the device. **(D)** COMSOL modeling and corresponding cell experiment for studying barotrauma as a result of applied transpulmonary pressure of 50 and 100 mbar. Scale: 1 mm.

Ventilator-induced lung injury (VILI) is thought to be closely related to strain heterogeneity in the lung, such that the injury occurs at high-strain regions (Beitler et al., 2016). The described fluidic-pneumatic platform is thus an ideal platform to model VILI. The two most commonly observed ventilator-induced lung injuries are volutrauma and barotrauma, which are closely related and result from alveolar overdistension caused by high tidal volumes and high transpulmonary pressure, respectively. To model volutrauma, the peak pressure of the air pressure waveform was increased from 50 to 100 mbar to generate increased concave volume and overdistension of the membrane. The concave volume for 50 and 100 mbar was 62.46 and 101.926 mm^3^, respectively. As evident from the live/dead assay for MLE-12 cell cultures under these conditions, the increased pressure and the associated overdistention caused cell death and/or detachment of the cells (Figure 5A). At higher pressures/volumes, the effect of spatial strain heterogeneity on cell number was amplified, as seen in the case of 100 mbar, where more cells were found to be at the edges (3 mm < r < 4 mm) than in the center (r < 3 mm). Among the two pressures imposed, the devices exposed to 50 mbar air pressure had higher cell numbers at the edges than those exposed to 100 mbar. Comparing the experimental observations with the corresponding strain derived from COMSOL simulation suggests that strains above ∼18% could be detrimental to the cells.

To model the lung transpulmonary pressure (alveolar air pressure—pleural fluid pressure) (Figure 5B), we have made a slight modification to the device to incorporate pleural fluid pressure. This was achieved by blocking the outlet of the media channels and replacing the syringe pump at the inlet with an open-ended syringe whose height can be easily varied (Figure 5C). The hydrostatic pressure developed in the chip by increasing the height of the syringe was used to mimic the pleural pressure, which applies a force on the membrane opposite to that of the air pressure, thus allowing us to define the transpulmonary pressure of the device as follows:

$$P_{TP}=P_{air}-P_{fluid}=\;P_{air}-\rho gh\;,$$ (4)

where P_TP_ is the transpulmonary pressure of the device, P_air_ is the applied peak pressure through the air pump, P_fluid_ is the hydrostatic pressure on the fluid side of the device, ρ is the density of the medium, g is the gravitational constant, and h is the height of the syringe above the device.

Leveraging this approach, we tested two conditions of varying transpulmonary pressure (50 and 100 mbar). To generate transpulmonary pressures of 50 mbar and 100 mbar, the syringe height was raised to 50 cm above the device height or kept at device height, respectively, while maintaining the applied air pressure at 100 mbar in both conditions. After 24 h of breathing, devices that were subjected to a transpulmonary pressure of 50 mbar showed minimal cell loss, despite the high air pressure encountered (Figure 5D). This is due to the hydrostatic pressure from the medium in the syringe opposing the high air pressure supplied from the air pump, which is akin to pleural fluid pressure opposing the high air pressure in the lung. Together these data highlight the ability of this biomechanically active platform to model VILI and its associated components and how such systems can be used as a predictive tool for determining regions that are predisposed to injury when subjected to mechanical ventilation.

### Device Supports Human Alveolar Cell Cultures

**FIGURE 6:**
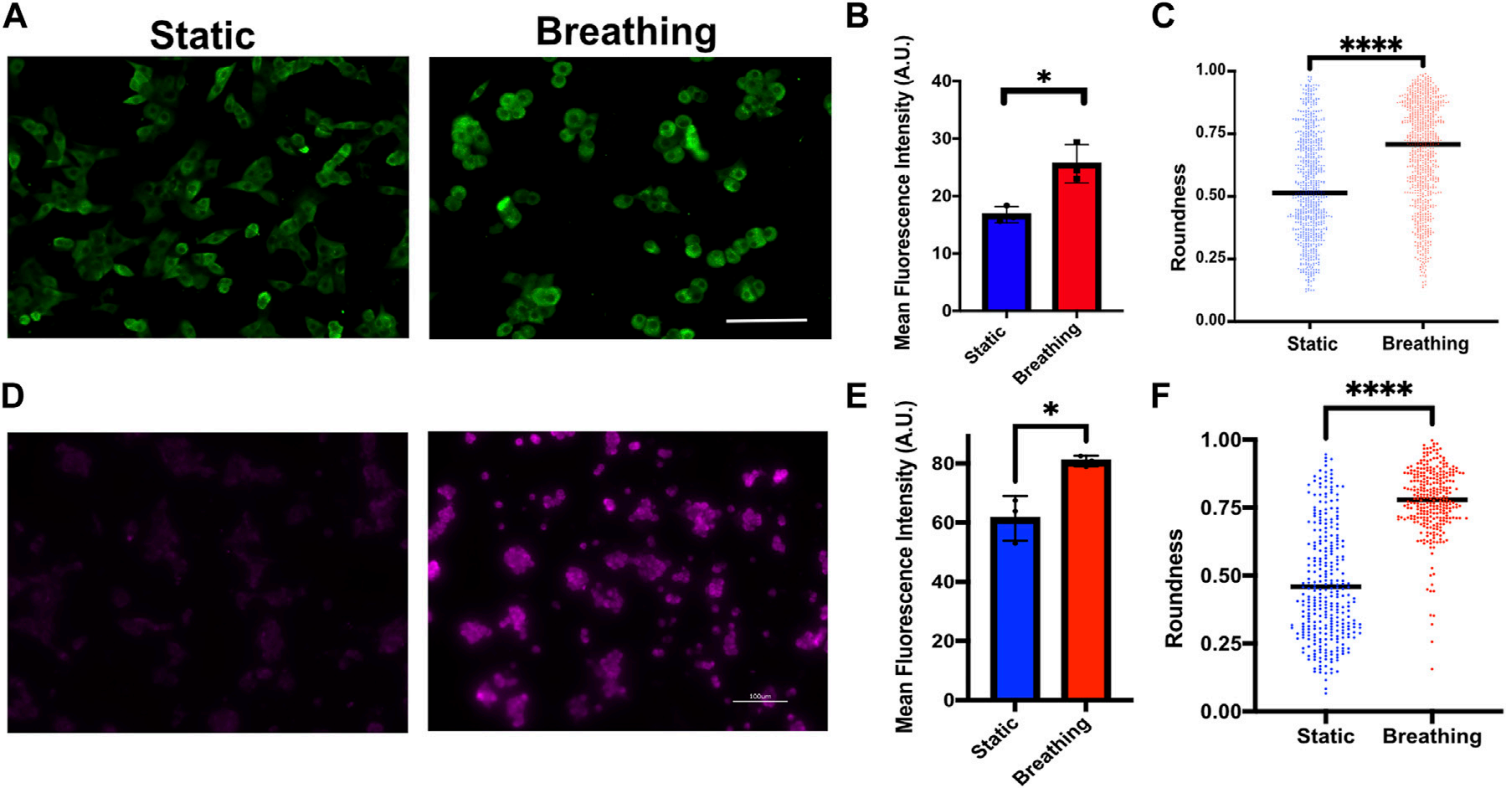
**(A)** Immunofluorescence staining of Pro-SPC (green) in primary human AT2 cells cultured under static and breathing conditions and **(B)** its quantification. N = 3 where each data point is an average of fluorescence intensity measurement from 8 to 15 images per independent device. **(C)** Quantification of cell morphology in static and breathing conditions. **(D)** Immunofluorescent staining of Pro-SPC (magenta) in iAT2 cells cultured under static and breathing conditions and **(E)** its quantification. N = 3 where each data point is an average of fluorescence intensity measurement from 9 to 13 images per independent device. **(F)** Quantification of cell morphology in static and breathing conditions. Scale: 100 μm.

Finally, we examined the potential of the platform to support human primary alveolar epithelial type 2 (hAT2) cells and human iPSC-derived alveolar type 2 (iAT2) cells. These cells were grown as alveolospheres in 3D cultures before dissociating them and seeding them onto the devices. Although the device can support long-term cultures of the cells (Supplementary Figure S7), hAT2 cells readily differentiate into alveolar type 1 (AT1) cells in 2D cultures and cease surfactant production. Therefore, the studies reported here were performed within 48 h of seeding the cells. The primary hAT2 cells were seeded at a density of 60,000 cells/cm^2^ and cultured on collagen type 1-coated membranes. The cells were exposed to breathing-like motion for 24 h and compared against the corresponding static cultures. We observed significantly higher levels of pro SP-C in cells that encountered breathing-like motions (Figures 6A, B). Additionally, we also observed distinct differences in cell morphology between the two conditions, wherein the cells that were exposed to breathing-like motions were significantly more rounded than cells in static conditions (Figure 6C). The roundness of the cell, which is defined as $\frac{4*area}{\pi *major\;axis^{2}}$ , was uniform across the device cultured in the static condition and no difference was observed between the center and edge of the membrane. On the contrary, the cells that were exposed to breathing-like motions displayed a significant difference between the edge and the center of the membrane. The cells at the center of the membrane were significantly more rounded than those at the edges, likely due to the spatially heterogeneous strain profile in the two regions (Supplementary Figure S8). We observed similar results with iAT2 cells, where cells cultured under breathing-like movements displayed significantly higher expression of pro-SPC as than static cultures (Figures 6D, E). As observed with primary hAT2 cells, iAT2 displayed more rounded morphologies in breathing-like conditions as than cells in static conditions (Figure 6F), although spatial differences in morphology were not observed in these cells. These results highlight the ability of culturing human alveolar epithelial cells within the device, a more clinically relevant cell source to model tissue-specific functions.

## Conclusion

This study describes the development of a microfluidic–pneumatic platform consisting of pneumatic and fluidic chambers separated by a thin membrane that supports alveolar epithelial cell culture. The device utilizes out-of-plane stretching of the membrane *via* cyclic air pressurization to mimic strain heterogeneity experienced during alveolar expansion due to breathing, thus allowing us to study the effect of strain heterogeneity within a single device.

Our results show that breathing-like motions and micromechanics in the local environment had a significant effect on cell morphology and critical functions of alveolar epithelial cells, such as surfactant production. We also utilized this platform to model VILI by incorporating transpulmonary pressure. One of the limitations of the current study is that the dimensions of the membrane used are much greater than alveolar size, which can prevent the cells from feeling curvature at a cellular level during stretch (Nossa et al., 2021). The length scale of the alveolar architecture will have a significant effect on the strain heterogeneity generated from breathing-like motions. Recent studies have recapitulated alveolar size and curvature within lung-on-chip systems (Huang et al., 2021; Zamprogno et al., 2021), although further studies are needed to characterize heterogeneous strain and its effect on cells within such systems mimicking the alveolar architecture. While our device incorporates only the epithelial cells in its current configuration, our approach provides a framework for developing more complex systems that capture alveolar micromechanics and spatial strain heterogeneity. The incorporation of other distal lung cells such as fibroblasts within these devices will facilitate the study of progressive changes in lung biomechanics in diseases involving heterotypic cell–cell interactions such as pulmonary fibrosis. Replacing the non-porous PDMS membrane used in this study with porous membranes would allow cell–cell interactions, while also enabling air–liquid interface culture (Huh et al., 2010; Stucki et al., 2018; Doryab et al., 2021a, Doryab et al., 2021b; Huang et al., 2021; Zamprogno et al., 2021). Finally, although we have only modeled positive pleural pressure by raising the height of the syringe, negative pleural pressure can be similarly modeled by lowering the syringe height below the device height and dynamic pleural pressure changes by using an automated system that adjusts the height of the syringe in concert with the air pressure. Apart from providing insights into the role of biomechanics on lung function, these platforms can also potentially serve as important predictive tools to prevent lung injuries or for drug screening (Artzy-Schnirman et al., 2021).

## Data Availability

The raw data supporting the conclusions of this article will be made available by the authors, without undue reservation.
