# Supplementary material for: An In Vitro Microfluidic Alveolus Model to Study Lung Biomechanics
Source: Front Bioeng Biotechnol. 2022 Feb 18;10:848699. doi: 10.3389/fbioe.2022.848699 (PMC8895303; doi:10.3389/fbioe.2022.848699)
Supplement: Supplementary file 3 [file DataSheet1.docx]

Supplementary Material for

**An *in vitro* microfluidic alveolus model to study lung biomechanics**

**Kumar et al.**

## Supplementary Figures

**Supplementary Figure 1.**


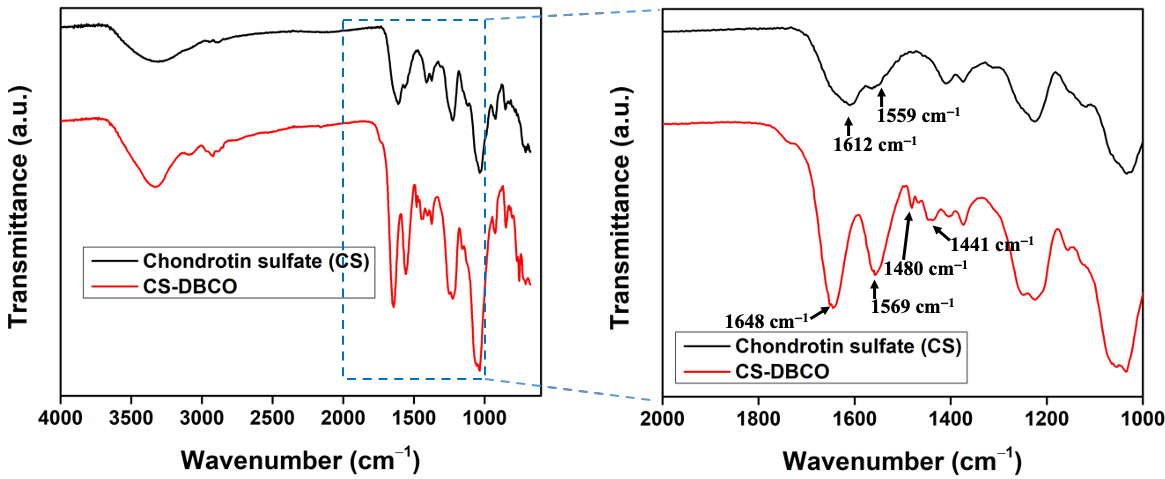


**Supplementary Figure 1.** ATR-FTIR spectra of the polymers CS and CS-DBCO. The arrow at 1648 cm^-1^ and 1569 cm^-1^ for amide-I (C=O stretching) and amide-II (N−H bending) respectively. The arrows at 1480 cm^-1^ and 1441 cm^-1^ are for the C=C stretching of the aromatic ring of CS-DBCO.

**Supplementary Figure 2.**
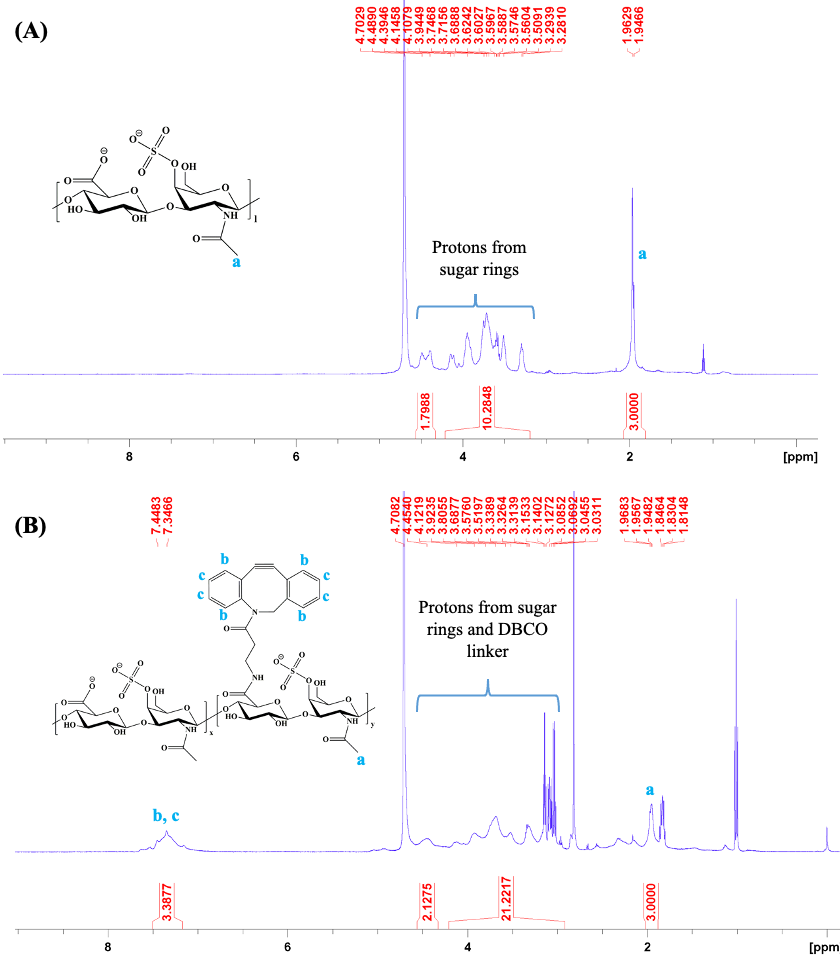


**Supplementary Figure 2.** ^1^HNMR spectra of the polymers recorded in D_2_O at room temperature. (A) NMR spectrum of chondroitin sulfate; (B) NMR spectrum of DBCO modified chondroitin sulfate.

**Supplementary Figure 3.**


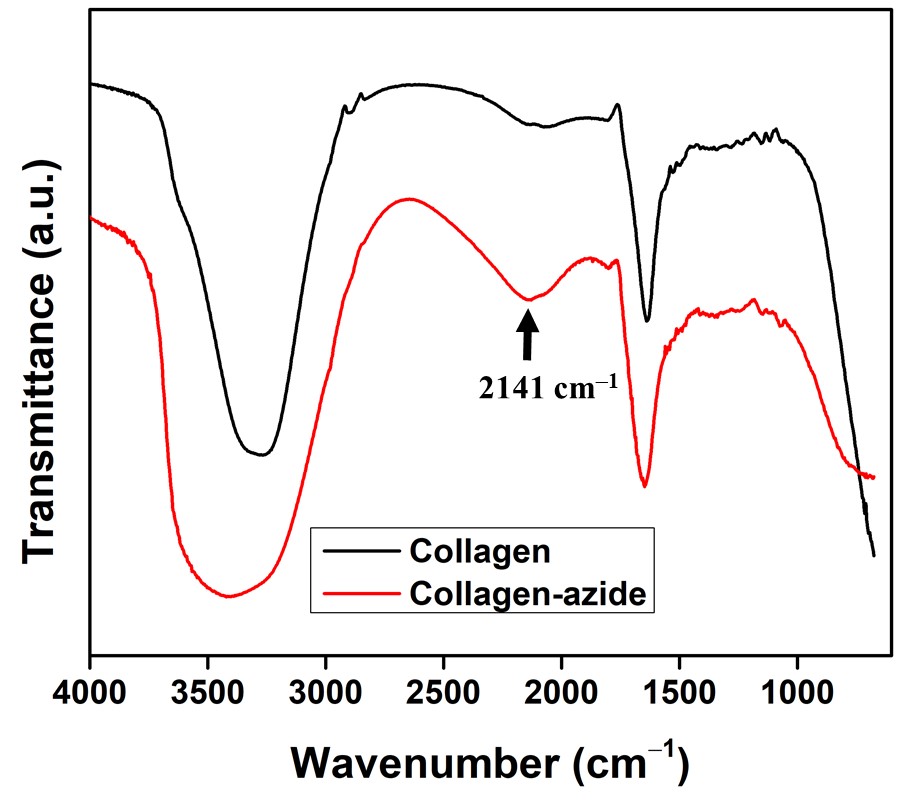


**Supplementary Figure 3.** ATR FTIR spectra of collagen and collagen-azide. The arrow indicates peak at 2141 cm^-1^ from the N≡N of azide group.

**Supplementary Figure 4.**


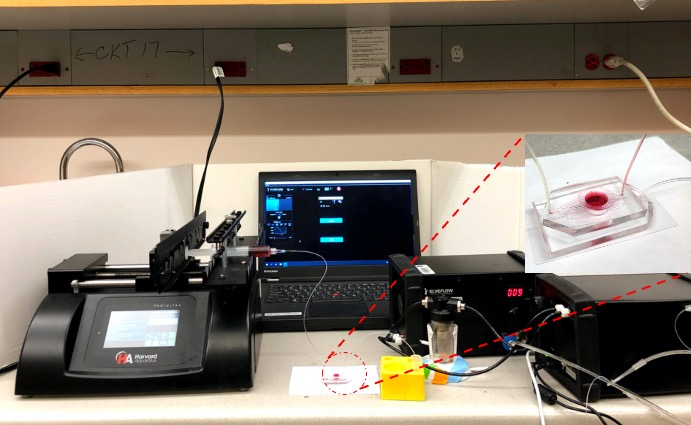


**Supplementary Figure 4**. Image of the setup showing connections to syringe pump (left) and air pump (right) programmed using the computer (center back). During the experiments, the device was placed inside an incubator. The cell culture chamber within the device in the image is perfused with red colored-fluid for visualization.

**Supplementary Figure 5.**

**Supplementary Figure 5.** ELISA for SP-A secreted by H441 cells cultured under static and breathing-like motions.

**Supplementary Figure 6.**

**
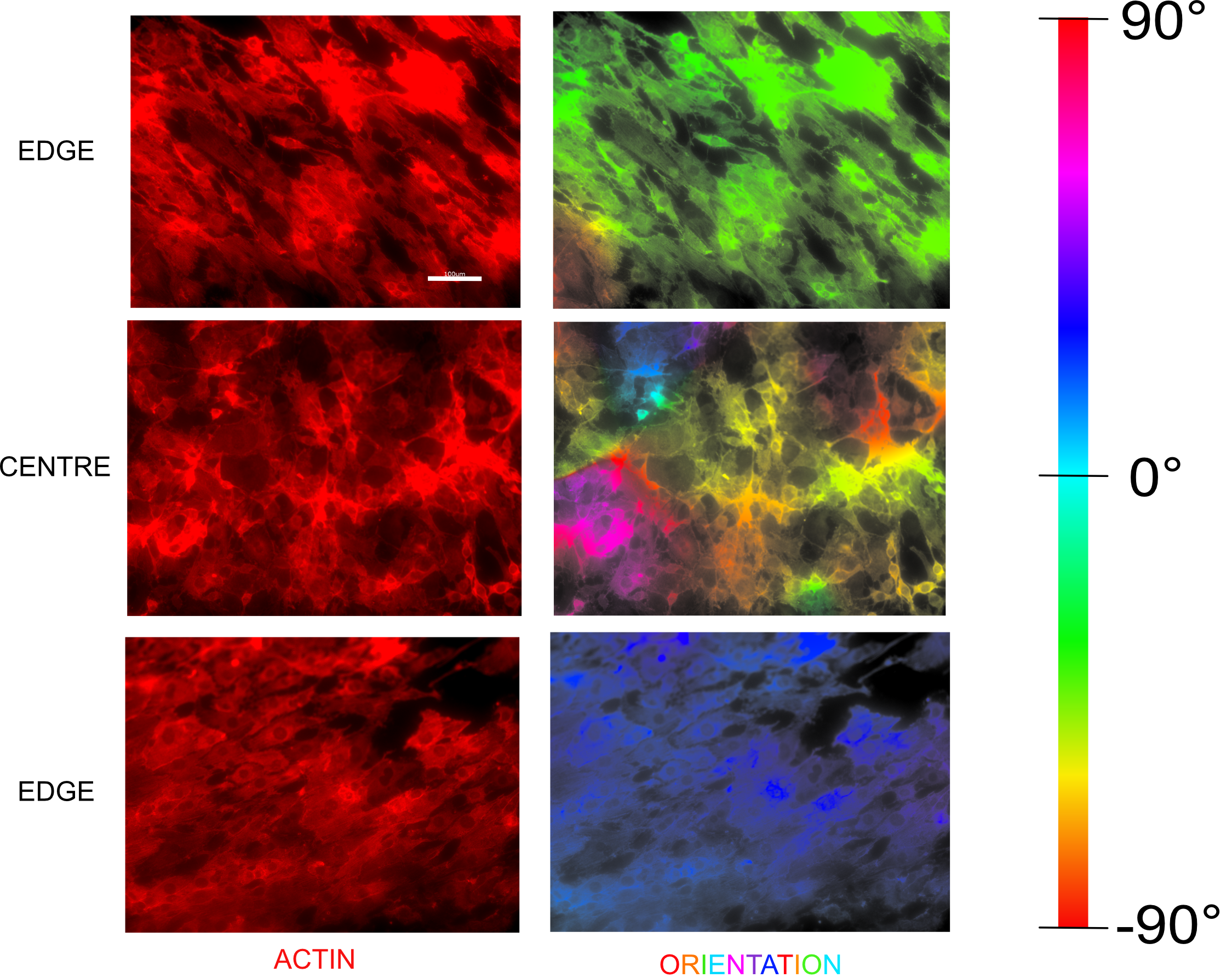
**

**Supplementary Figure 6.** Cellular alignment of 3T3 fibroblasts cultured within the device and exposed to breathing-like motions. The cells were stained with phalloidin to visualize (first column) and analyzed for alignment using ImageJ (second column). The color of the cells in the second column corresponds to their angle of orientation. Scale: 100 μm

**Supplementary Figure 7.**


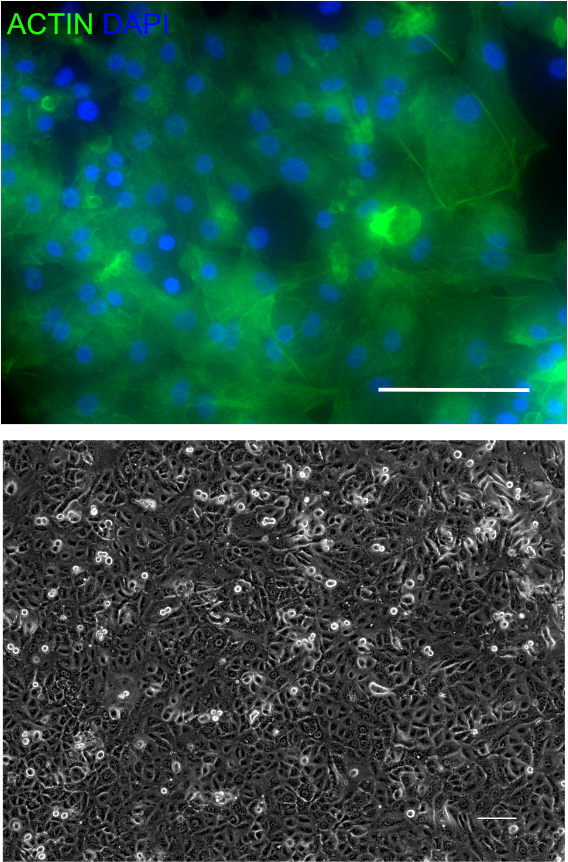


**Supplementary Figure 7.** Top**:** Phalloidin and nucleus staining for adult human AT2 cells cultured within the device for 12 days. Bottom: Phase contrast image of H441 cells cultured in the device for 10 days. Scale: 100 μm

**Supplementary Figure 8.**

**Supplementary Figure 8.** Difference in cell morphology of adult human AT2 cells cultured under static and breathing-like motions. While static cultures showed no spatial differences, those exposed to breathing-like motions have cells with more rounded shape at the center than at the edges.

**Supplementary Table 1.**

**Mechanical properties of PDMS membrane:**

| **Property** | **Value** | **Reference** |
| --- | --- | --- |
| **Young’s Modulus** | **~1000 kPa** | (Liu et al., 2009) |
| **Mooney Rivlin constants**  **C10**  **C01** | **75.5 kPa**  **5.7 kPa** | (Yoon et al., 2010) |

**Supplementary Table 1.** Mechanical properties of PDMS (10:1::base:crosslinker) membrane. The Mooney-Rivlin constants were used for computational analyses.

**References:**

Liu, M., Sun, J., Sun, Y., Bock, C., and Chen, Q. (2009). Thickness-dependent mechanical properties of polydimethylsiloxane membranes. *J. Micromechanics Microengineering* 19, 35028. doi:10.1088/0960-1317/19/3/035028.

Yoon, S., Reyes-Ortiz, V., Kim, K., Seo, Y. H., and Mofrad, M. R. K. (2010). Analysis of Circular PDMS Microballoons With Ultralarge Deflection for MEMS Design. *J. Microelectromechanical Syst.* 19, 854–864. doi:10.1109/JMEMS.2010.2049984.
